# The need to set explicit goals for human germline gene editing public dialogues

**DOI:** 10.1007/s12687-024-00710-1

**Published:** 2024-05-08

**Authors:** Wendy P. Geuverink, Diewertje Houtman, Isabel R. A. Retel Helmrich, Sophie van Baalen, Britta C. van Beers, Carla G. van El, Lidewij Henneman, Michelle D. Kasprzak, Danielle Arets, Sam R. Riedijk, the ‘De DNA dialogen’ consortium

**Affiliations:** 1 Department of Human Genetics, Amsterdam UMC, Location Vrije Universiteit Amsterdam, Amsterdam, the Netherlands; 2 Amsterdam Reproduction and Development Research Institute, Amsterdam, the Netherlands; 3 Erasmus Medical Center, Department of Clinical Genetics, Rotterdam, the Netherlands; 4 Rathenau Institute, The Hague, the Netherlands; 5 Faculty of Law, Department of Legal Philosophy, Vrije Universiteit, Amsterdam, the Netherlands; 6 Amsterdam Public Health Research Institute, Amsterdam, the Netherlands; 7 Willem de Kooning Academy, Rotterdam University of Applied Sciences, Rotterdam, the Netherlands; 8 Readership Journalism & Innovation, Fontys University of Applied Sciences, Tilburg, the Netherlands

**Keywords:** Human Germline Gene Editing, Public Engagement, Societal Alignment, Open Science

## Abstract

Given the potentially large ethical and societal implications of human germline gene editing (HGGE) the urgent need for public and stakeholder engagement (PSE) has been repeatedly expressed. However, the explicit goals of such PSE efforts often remain poorly defined. In this program report, we outline the goals of our Dutch project called *De DNA dialogen* (The DNA dialogues). We believe that setting explicit goals in advance is essential to enable meaningful PSE efforts. Moreover, it enables the evaluation of our engagement efforts. The following four goals, which result from intensive consultations among the transdisciplinary projects’ consortium members and based on the literature, form the foundation for how we will engage the public and stakeholders in deliberation about HGGE: 1) Enable publics and stakeholders to deliberate on “what if” questions, before considering “whether” and “how” questions regarding HGGE, 2) Investigate agreement and disagreement in values and beliefs regarding HGGE in order to agree and disagree more precisely, 3) Involve diverse publics with various perspectives, with a focus on those that are typically underrepresented in PSE, 4) Enable societally aligned policy making by providing policymakers, health care professionals and legal experts insight into how values are weighed and ascribed meaning in the context of HGGE by various publics, and how these values relate to the principles of democratic rule of law and fundamental rights. The effort to describe our goals in detail may serve as an example and can inform future initiatives striving for open science and open governance in the context of PSE.

**Supplementary Information:**

The online version contains supplementary material available at 10.1007/s12687-024-00710-1.

## Introduction

Human germline gene editing (HGGE) introduces heritable genetic alterations to the human germline and has the potential to impact clinical practice by providing a way to genetically remove severe genetic conditions or modify traits in the embryos of prospective parents (Ormond et al. 2017). One of the frequently mentioned considerations regarding HGGE is that it will change the human gene pool as soon as genetically edited children grow up and reproduce as germline genetic modifications are heritable across generations. Editing the human germline raises many fundamental questions that are not only relevant for individuals and their offspring, but also for society and even humanity (International Bioethics Committee 2015; Council of Europe 1997; Lander et al. 2019). The potentially large ethical and societal implications urge deliberation about HGGE based on input provided from the scientific community as well as from patients and the public at large (Scheufele et al. 2021; Iltis et al. 2021). The urgent need for public and stakeholder engagement (PSE) on HGGE has repeatedly been expressed by various authoritative (inter)national bodies and committees (Lander et al. 2019; WHO Expert Advisory Committee on Developing Global Standards for Governance and Oversight of Human Genome Editing 2021; de Wert et al. 2018; Chan et al. 2015; Nuffield Council on Bioethics 2018; National Academy of Medicine 2020; Commissie Genetische Modificatie (COGEM) and Gezondheidsraad 2017; National Academies of Sciences 2017; Baltimore et al. 2015). The 2023 statement by the Organizing Committee of the Third International Summit on Human Genome Editing underscores that the societal discussion and policy debate is far from conclusion (https://royalsociety.org/news/2023/03/statement-third-international-summit-human-genome-editing/).

In the Netherlands, we have been responding to these urgent global calls for PSE about HGGE by organizing public dialogues. The current project called *De DNA dialogen* (The DNA dialogues; 2022–2026) follows the previous DNA dialogue project, which was conducted from 2019–2021 and engaged various publics by organizing 27 dialogues about HGGE in the Netherlands (van Baalen et al. 2021). Although this former project involved a variety of publics, some groups were not explicitly engaged. Public acceptance was mapped, as well as the main values related to HGGE that came up during dialogue (van Baalen et al. 2021; Houtman et al. 2022). The identified values were important for *acceptance* and for protection in *practical application* and *in society* (van Baalen et al. 2021). Now, more in-depth knowledge, for example about the weighing of values in the context of HGGE and how these relate to international and national legal frameworks is necessary for political deliberation with the aim of societal alignment (Ribeiro et al. 2018). In the context of our Dutch representative democracy, societal alignment means that a broad variety of perspectives in society is represented, so that governments at national and European level, as well as policy makers in professional societies, could integrate these perspectives in their policy development.

In *De DNA dialogen* (2022–2026) we strive to engage a broad range of publics with the specific aim to involve those typically underrepresented in PSE (Table 1). Moreover, we will go beyond arguments related to HGGE, and delve into people's understanding of these arguments, the underlying values, how these values are contextualized by individuals, and how these values relate to existing and possible systems of governance. Based on our previous experiences with DNA dialogues, we learned that space for emotional and moral arguments is crucial in widening and deepening the dialogue around germline modification.

**Table 1:**
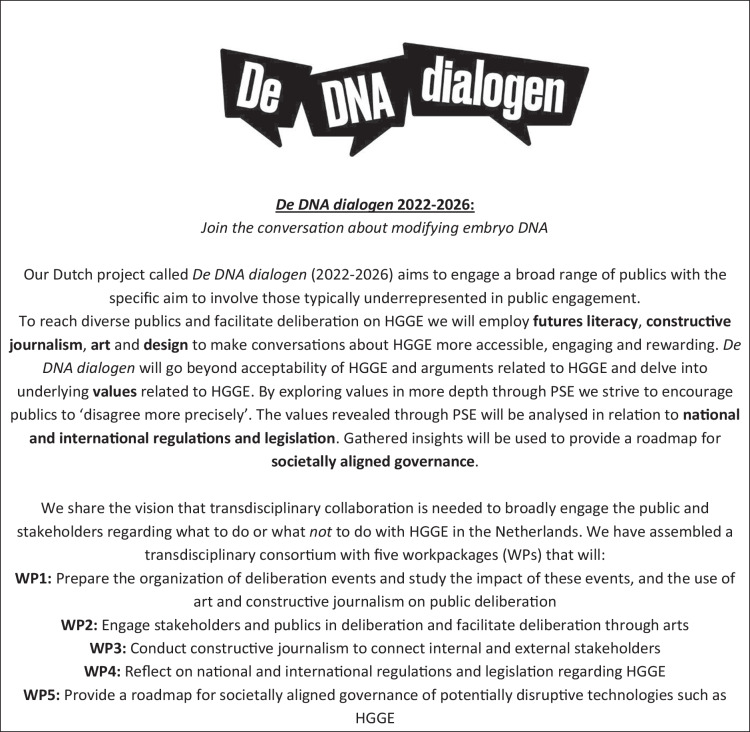
*De DNA dialogen* project outline

While there is much agreement on the importance of PSE on HGGE, there is less agreement on the goals of such PSE (Iltis et al. 2021; Scheufele et al. 2021; Baylis 2017). There are for example different perspectives on educating the public as a precondition or goal of PSE and whether PSE should strive for consensus or not (Scheufele et al. 2021; Simis et al. 2016; Baylis et al. 2020).

**Fig. 1:**
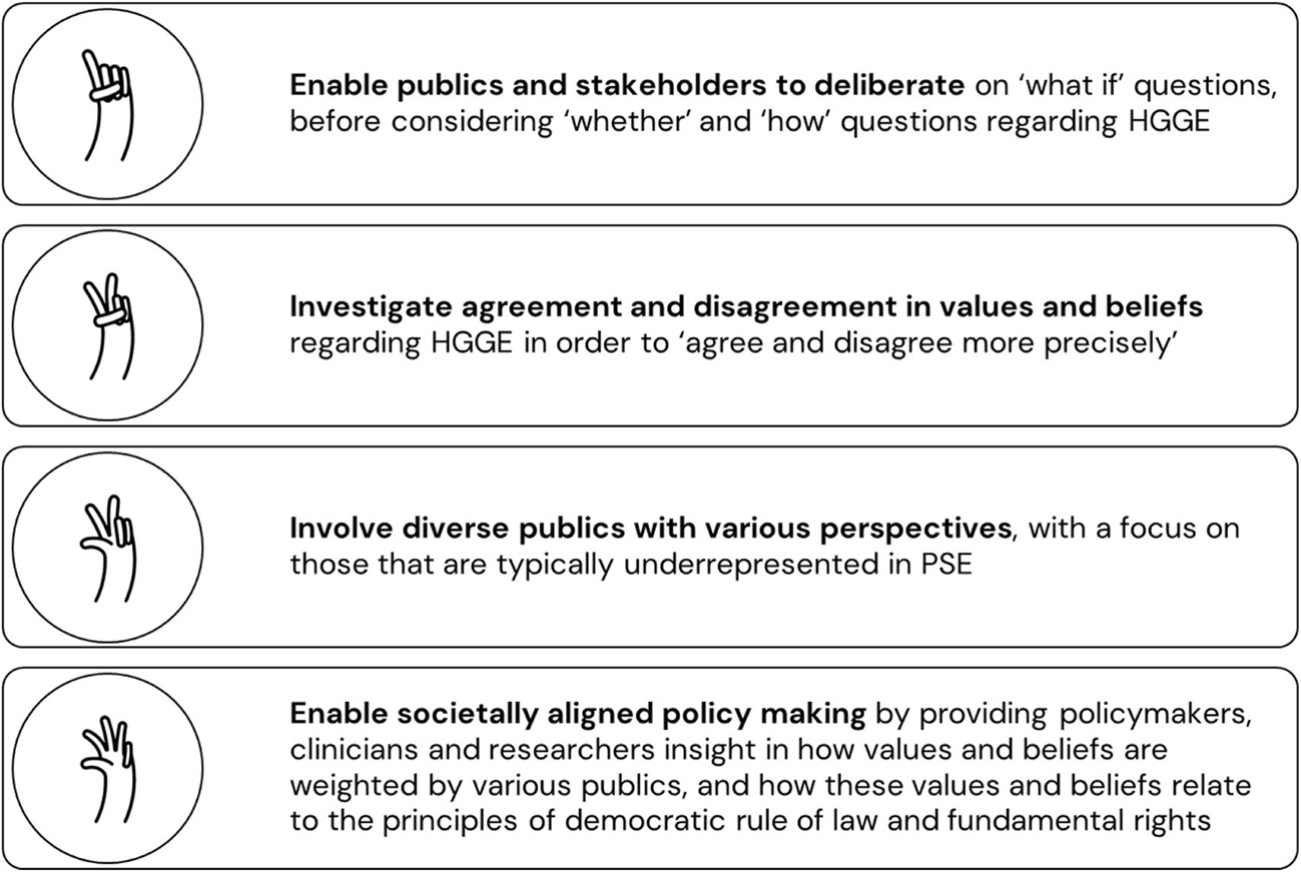
Public and Stakeholder Engagement Goals in *De DNA dialogen*

In *De DNA dialogen,* our consortium has formulated four overarching goals that form the foundation for how we will engage various publics and stakeholders on HGGE in the Netherlands (Fig. 1). By describing the four goals in this paper we aim to enable open science and more precise comparisons with other PSE projects in the future.

## Goal 1: Enable publics and stakeholders to deliberate

A classic understanding of deliberation is that it is used to resolve problematic situations through the process of exchanging competing reasons, with the goal of making informed and reasoned decisions (Habermas 2001). The yield of such deliberation typically may be shaping policy or making recommendations. However, the idea that a clash of rational arguments will lead to the best outcome or decisions is criticized, especially when it comes to highly complex dilemmas involved in technological developments (Pellizzoni 2001), such as HGGE. A growing body of recent research shows that many of our rational considerations are fed and supported by our emotions and vice versa (Roeser and Pesch 2016; Roeser et al. 2020; van Doorn 2023). Therefore, a more relational approach of deliberation with more elements from dialogue theory in which inquiry dynamics such as exploring, co-creating shared meaning, sharing feelings and building understanding is needed (Escobar 2009). In our project we aim to combine the inquiry dynamics that lead to hearing a wide variety of voices, values and perspectives with the more classic advocacy dynamics of the deliberation process, such as exchanging reasons and being inclusive of all members (Scheufele et al. 2021; Schneiderhan and Khan 2008; Escobar 2011). Based on the above considerations and the various definitions of deliberation cited in the literature, we define deliberation as follows: deliberation is a multi-way communication among the public and/or stakeholders whereby a conversation starter (e.g. scenario, statement, visual, question) is presented, followed by facilitated discussion in which people are willing to engage in both emotional and rational reflection on a multifaceted issue (here: HGGE). This requires participants’ willingness to both listen to and consider opposing viewpoints on the topic (Scheufele et al. 2021; Iltis et al. 2021; Schneiderhan and Khan 2008). When successful deliberation occurs, it is expected that the generated public insights—that might otherwise be unavailable to elected representatives or policy makers- will enrich and improve policy outcomes (Sirianni 2010).

As previously described elsewhere, we recommend starting the deliberation on HGGE with "what if" questions that create time for the audience to reflect, slow down and use their imagination (Houtman et al. 2023). “What if” questions allow the audience to think about the ways HGGE could improve or problematize (their) lives and those of (their) future children, and what future scenarios would be (un)desirable and (in)consistent with their values. Considering these future scenarios also creates space to exchange values and perspectives. The questions “whether” and “how” thereafter provide valuable insight into how practice and policy around HGGE should be organized, which will become relevant once the “what if” scenarios, of what our society may become, have been deliberated on.

## Goal 2: Investigate agreement and disagreement in values and beliefs

After the first International Summit on Human Gene Editing in 2015 it was concluded that “It would be irresponsible to proceed with any clinical use of germline editing unless and until […] there is broad societal consensus about the appropriateness of the proposed application” (http://go.nature.com/2pwNV7w). The call for broad societal consensus provided, at the time, the scope to ask the fundamental question of whether HGGE should be used at all or, in the absence of broad societal consensus, should not be accepted (Almeida and Ranisch 2022; Jezierska 2019; Baylis 2017; Hurlbut 2019; Andorno et al. 2020). The debate on the meaning and importance of this envisaged broad societal consensus has subsequently been conducted extensively. International reports on the future of HGGE published since 2015 have seen a shift in which the call for broad societal consensus on HGGE and the question of its use per se is no longer as loud and seems to have been replaced by a call for clear and strict regulations and for conditions and objectives under which HGGE might be acceptable (Almeida and Ranisch 2022; Van Beers 2020; Greely 2019). In *De DNA dialogen* we believe that many different voices should be included in PSE regarding HGGE and that it is still relevant to ask the question of whether or not to apply HGGE. At the same time, we do not strive for consensus in the sense of unanimity or simple majority, but rather on recognizing and clarifying value differences (Mouffe 2013). In other words, we aim to agree and disagree more precisely. It is precisely the feelings of discomfort, of "not knowing", that need to be heard (Morrison and de Saille 2019). To do so, we strive to gain insight into how values and beliefs are weighed and ascribed meaning in the context of HGGE by various publics. This is especially important in case of potentially disruptive and complex technologies such as HGGE that may have far-reaching societal implications which call for governance and regulatory structures that take into account a variety of perspectives (Scheufele et al. 2021).

## Goal 3: Involve diverse publics with various perspectives

Various national and international bodies and committees emphasize the importance of involving the public in broad societal dialogue. They state that PSE should actively involve those who may (likely) be impacted (e.g., persons with a genetic disorder, their parents and families, couples at risk of having a child with a severe genetic disorder and people with reproductive stakes) and those affected by inequities (Nuffield Council on Bioethics 2018; Commissie Genetische Modificatie (COGEM) and Gezondheidsraad 2017). In *De DNA dialogen,* we work with the following definition of diversity: “Cultural differences in values, beliefs, and behaviors learned and shared by groups of interacting people defined by nationality, ethnicity, gender, age, physical characteristics, sexual orientation, economic status, education, profession, religion, organizational affiliation, and any other grouping that generates identifiable patterns” (Bennett and Bennett 2004).

PSE generally involves a relatively homogenous group of participants, namely persons that are highly educated, knowledgeable on the topic, predominantly white, affluent, and have relatively high trust in science (Humm, Schrögel, and Leßmöllmann 2020; Kennedy et al. 2018; Pandya 2012). We aim to involve diverse publics with various perspectives, and we specifically aim to engage those typically underrepresented in PSE, such as people with functional impairment or people who have low trust in science. Therefore, focused engagement efforts will be needed. Inclusivity of PSE is crucial as excluding groups or persons with their own perspectives and lived experiences means that these may not be represented. This underrepresentation of perspectives poses a threat to the development of societally aligned governance of HGGE. To reach diverse publics and facilitate deliberation we will employ interactive art and design (i.e., fine art, theater, music, photography). Moreover, we will work with key figures in communities to teach us how our engagement efforts may feed into the motives and needs of specific communities resulting in accessible and engaging conversations about HGGE. In addition, these collaborations can establish reciprocal relationships of trust between our consortium and engaged communities, because these key figures have a close connection with the communities and share their values (e.g., a Dutch Christian patient organization). Although during the dialogue activity itself certain shared goals are required, such as listening to other perspectives and being curious, we recognize that the overarching goals that we as a consortium pursue need not be shared by our participants. Those publics that we aim to engage may not be motivated to participate in dialogue to pursue societal alignment for HGGE. For some, a motive to participate in our PSE may be to feel connected and heard, whereas others may feel rewarded if they go home with an exciting experience. We are aware that not everybody will feel gratified in a similar way from participating. Together with social partners we aim to find out about the needs and interests of the target group and align our dialogue strategies with those. In this way we aim to avoid becoming the researchers who, in the eyes of citizens, only come to get something without giving anything back.

## Goal 4: Enable societally aligned policy making

Increasingly, also in PSE about HGGE, goals of science communication are moving from educating the public to consulting, involving, and even collaborating with publics (Burall 2018; Dryzek et al. 2020; Houtman, Vijlbrief, and Riedijk 2021; Andorno et al. 2020). Instead of being educated, the goal of PSE becomes to generate a shared understanding of the topic, issues, and perspectives. For PSE to be effective and meaningful it must be consequential. In other words, how do we translate the needs and values of the public into policy development? In representative democracies, as in the Netherlands and across Europe, decisions concerning the regulation of HGGE are in the hands of elected politicians. Therefore, we cannot directly feed the outcomes of the dialogues (i.e., public values) into national or international policies. Instead, we aim to support elected politicians to make careful and well-informed policy decisions. This entails more than simply “informing the policy environment” about the outcomes of the dialogues (Baylis 2019). We aim to stimulate the debate among policymakers about values related to HGGE; i.e., both public values originating from the dialogues and values originating from their political ideologies and interests. However, policymakers will not only want to be informed about the outcomes of public dialogue on HGGE and the public values that emerge in that process but will also inevitably have to consider various possible regulatory models in order to develop policies in this field based on these outcomes and values. Our approach will therefore also provide policymakers with an overview and analysis of various regulatory models. Presenting policy makers with different options for and reflection on regulatory models for the governance of HGGE can help with finding ways to operationalize the alignment of policy, laws and regulations with the values and needs of the public.

Societal alignment will not take place in a legal vacuum. We consider the relation between law and PSE a two-way street, that is, as part of a circular dynamic: law is not only the outcome of political decision making or legal translation of policy making, but, conversely, law also feeds into decision- and policy making, for example in the form of fundamental rights and legal principles. Given the rule of law, policy making informed by the outcomes of PSE will need to be in line with fundamental rights law (Van Beers 2020; Commissie Genetische Modificatie (COGEM) and Gezondheidsraad 2017; Van Est et al. 2016) as laid down not only in the Dutch Constitution, but also in international and European human rights law. For example, how do the outcomes of PSE compare to various legal interpretations of the principle of human dignity or the right to reproduction, which both have been recognized in European human rights case law? Moreover, to contribute to policy making, it is important to examine how the values and future scenarios that emerge from PSE relate to imaginaries and values underpinning current legal approaches to HGGE (Jasanoff 2015; Klink et al. 2016). The law is after all not just a collection of commands backed up by coercion, but it also tells a certain story and offers an “imaginary” to interpret the world. Legal concepts set the stage for further debate and bring various values to expression. We refer to this as the communicative and expressive functions of the law (Klink et al. 2016; Van Beers 2015; Poort et al. 2016). In addition, various possible regulatory models need to be explored to examine which of these models can possibly do justice to the values, and future scenarios resulting from PSE. These regulatory models may include prohibitions, moratoria, professional self-regulation, regulation through an administrative agency and ‘interactive legislation’(Van der Burg and Brom 2000).

## In conclusion

*De DNA dialogen*, provides an opportunity to involve the public ‘to participate thoughtfully in imagining the futures we want and governing technological change accordingly’ (Jasanoff et al. 2015). Specifying our goals for PSE has resulted in a shared understanding as well as a common language to communicate about our goals, as a consortium. This is particularly valuable within the setting of our transdisciplinary consortium, in which essential terms may carry a different meaning. We highly recommend future initiatives striving for open science in the context of PSE about HGGE or other potentially disruptive technologies to co-create and share their common language, to make their work open access and enable comparison with similar PSE initiatives.

### Supplementary Information

Below is the link to the electronic supplementary material.

Supplementary file1 (DOCX 14.3 KB)

## References

[CR1] Almeida M, Ranisch R (2022). Beyond safety: mapping the ethical debate on heritable genome editing interventions. Human Social Sci Commun.

[CR2] Andorno R, Baylis F, Darnovsky M, Dickenson D, Haker H, Hasson K, Lowthorp L, Annas GJ, Bourgain C, Drabiak K (2020) Geneva Statement on Heritable Human Genome Editing: The Need for Course Correction. Trends Biotechnol 10.1016/j.tibtech.2019.12.022 32014274

[CR3] Baltimore D, Baylis F, Berg P, Daley GQ, Doudna JA, Lander ES, Lovell-Badge R, Ossorio P, Pei D, Thrasher A (2015). On human gene editing: International summit statement.

[CR4] Baylis F (2017). Human germline genome editing and broad societal consensus. Nat Hum Behav.

[CR5] Baylis F (2019) Altered inheritance: CRISPR and the ethics of human genome editing. Harvard University Press

[CR6] Baylis F, Darnovsky M, Hasson K, Krahn TM (2020). Human germline and heritable genome editing: the global policy landscape. The CRISPR Journal.

[CR7] Beers V, Britta C (2015). Is Europe ‘giving in to baby markets?’Reproductive tourism in Europe and the gradual erosion of existing legal limits to reproductive markets. Med Law Rev.

[CR8] Bennett JM, Bennett MJ (2004) Developing intercultural sensitivity: An integrative approach to global and domestic diversity. na

[CR9] Burall S (2018). Rethink public engagement for gene editing. Nature.

[CR10] Chan S, Donovan PJ, Douglas T, Gyngell C, Harris J, Lovell-Badge R, Mathews DJH, Regenberg A, Group On Behalf of the Hinxton (2015) Genome editing technologies and human germline genetic modification: The Hinxton Group Consensus Statement. Am J Bioethics 15 (12): 42-4710.1080/15265161.2015.1103814 10.1080/15265161.2015.1103814 26632362

[CR11] Commissie Genetische Modificatie (COGEM), and Gezondheidsraad. 2017. Ingrijpen in het DNA van de mens, Morele en maatschappelijke implicaties van kiembaanmodificatie. COGEM (Bilthoven)

[CR12] Council of Europe (1997) Explanatory Report to the Convention for the protection of Human Rights and Dignity of the Human Being with regard to the Application of Biology and Medicine: Convention on Human Rights and Biomedicine. Council of Europe

[CR13] de Wert G, Pennings G, Clarke A, Eichenlaub-Ritter U, van El CG, Forzano F, Goddijn M, Heindryckx B, Howard HC, Radojkovic D, Rial-Sebbag E, Tarlatzis BC, Cornel MC (2018). Human germline gene editing: Recommendations of ESHG and ESHRE. Eur J Hum Genet.

[CR14] Dryzek JS, Nicol D, Niemeyer S, Pemberton S, Curato N, Bächtiger A, Batterham P, Bedsted B, Burall S, Burgess M (2020). Global citizen deliberation on genome editing. Science.

[CR15] Escobar O (2009) The dialogic turn: Dialogue for deliberation. In-Spire Journal of Law, Politics and Societies

[CR16] Escobar O (2011). Public dialogue and deliberation: A communication perspective for public engagement practitioners.

[CR17] Est V, Rinie JT, Kool L, Nijsingh N, Rerimassie V, Stemerding D (2016). Rules for the digital human park: Two paradigmatic cases of breeding and taming human beings: Human germline editing and persuasive technology.

[CR18] Greely HT (2019). How should science respond to CRISPR’d babies?. Issues Sci Technol.

[CR19] Habermas J (2001) "From Kant's" Ideas" of pure reason to the" Idealizing" presuppositions of communicative action: Reflections on the detranscendentalized" Use of Reason." Pluralism and the pragmatic turn: The transformation of critical theory: 11–39

[CR20] Houtman D, Geuverink W, Helmrich IRAR, Vijlbrief B, Cornel M, Riedijk S (2023) What if” should precede “whether” and “how” in the social conversation around human germline gene editing. J Commun Gen: 1–5 10.1007/s12687-023-00652-0 PMC10444910 37326787

[CR21] Houtman D, Vijlbrief B, Polak M, Pot J, Verhoef P, Cornel M, Riedijk S (2022) Changes in opinions about human germline gene editing as a result of the Dutch DNA-dialogue project. Eur J Human Gen 1–8. 10.1038/s41431-022-01114-w 10.1038/s41431-022-01114-w PMC9095815 35551502

[CR22] Houtman D, Vijlbrief B, Riedijk S (2021) Experts in science communication: A shift from neutral encyclopedia to equal participant in dialogue. EMBO reports e52988 10.15252/embr.202152988 PMC8344925 34269513

[CR23] Humm C, Schrögel P, Leßmöllmann A (2020) Feeling left out: underserved audiences in science communication. Media Commun 8 (1): 164–176. 10.17645/mac.v8i1.2480

[CR24] Hurlbut JB (2019). Human genome editing: ask whether, not how. Nature.

[CR25] Iltis AS, Hoover S, Matthews KRW (2021). Public and Stakeholder Engagement in Developing Human Heritable Genome Editing Policies: What Does it Mean and What Should it Mean?. Front Politic Sci.

[CR26] International Bioethics Committee (2015) Report of the IBC on updating its reflection on the Human Genome and Human Rights*.* United Nations Educational, Scientific and Cultural Organization (Paris). https://unesdoc.unesco.org/ark:/48223/pf0000233258

[CR27] Jasanoff S (2015) Future imperfect: Science, technology, and the imaginations of modernity. Dreamscapes of modernity: Sociotechnical imaginaries and the fabrication of power 1–33

[CR28] Jasanoff S, Benjamin Hurlbut J, Saha K (2015). CRISPR democracy: Gene editing and the need for inclusive deliberation. Issues Sci Technol.

[CR29] Jezierska K (2019) With habermas against habermas. Deliberation without consensus. J Deliber Democ 15 (1)

[CR30] Kennedy EB, Jensen EA, Verbeke M (2018). Preaching to the scientifically converted: evaluating inclusivity in science festival audiences. Intl J Sci Educ, Part B.

[CR31] Lander ES, Baylis F, Zhang F, Charpentier E, Berg P, Bourgain C, Friedrich B, Joung JK, Li J, Liu D, Naldini L, Nie JB, Qiu R, Schoene-Seifert B, Shao F, Terry S, Wei W, Winnacker EL (2019). Adopt a moratorium on heritable genome editing. Nature.

[CR32] Morrison M de Saille S (2019) CRISPR in context: towards a socially responsible debate on embryo editing. Palgrave Commun 5 (1)

[CR33] Mouffe C (2013) Agonistics: Thinking the world politically. Verso Books

[CR34] National Academies of Sciences, Engineering, and Medicine (2017) Human Genome Editing: Science, Ethics, and Governance. (Washington, DC: The National Academies Press). https://nap.nationalacademies.org/catalog/24623/human-genome-editing-science-ethics-and-governance 28796468

[CR35] National Academy of Medicine, National Academy of Sciences, and the Royal Society (2020). *Heritable Human Genome Editing.* (Washington, DC: The National Academies Press). https://nap.nationalacademies.org/catalog/25665/heritable-human-genome-editing 32897669

[CR36] Nuffield Council on Bioethics. 2018. Genome Editing and Human Reproduction: social and ethical issues. (London: Nuffield Council on Bioethics). https://nap.nationalacademies.org/catalog/25665/heritable-human-genome-editing

[CR37] Ormond KE, Mortlock DP, Scholes DT, Bombard Y, Brody LC, Faucett WA, Nanibaa’A G, Hercher L, Isasi R, Middleton A (2017) Human germline genome editing. Am J Human Gen 101 (2): 167-176 10.1016/j.ajhg.2017.06.012 10.1016/j.ajhg.2017.06.012 PMC5544380 28777929

[CR38] Pandya RE (2012). A framework for engaging diverse communities in citizen science in the US. Front Ecol Environ.

[CR39] Pellizzoni L (2001). The myth of the best argument: Power, deliberation and reason1. Br J Sociol.

[CR40] Poort L, Van Beers B, Van Klink B (2016) Introduction: symbolic dimensions of biolaw. In Symbolic legislation theory and developments in biolaw, 1–15. Springer

[CR41] Ribeiro B, Bengtsson L, Benneworth P, Bührer S, Castro-Martínez E, Hansen M, Jarmai K, Lindner R, Olmos-Peñuela J, Ott C (2018). Introducing the dilemma of societal alignment for inclusive and responsible research and innovation. J Respons Innov.

[CR42] Roeser S, Pesch U (2016). An emotional deliberation approach to risk. Sci Technol Human Values.

[CR43] Roeser S, Taebi B, Doorn N (2020). Geoengineering the climate and ethical challenges: What we can learn from moral emotions and art. Crit Rev Int Soc Pol Phil.

[CR44] Scheufele DA, Krause NM, Freiling I, Brossard D (2021). What we know about effective public engagement on CRISPR and beyond. Proc Natl Acad Sci.

[CR45] Schneiderhan E, Khan S (2008). Reasons and inclusion: The foundation of deliberation. Sociol Theory.

[CR46] Simis MJ, Madden H, Cacciatore MA, Yeo SK (2016) The lure of rationality: Why does the deficit model persist in science communication? Public Understand Sci 25 (4): 400–414. 10.1177/0963662516629749. https://journals.sagepub.com/doi/abs/10.1177/0963662516629749. 10.1177/0963662516629749 27117768

[CR47] Sirianni C (2010) Investing in democracy: Engaging citizens in collaborative governance. Rowman & Littlefield

[CR48] van Baalen S, Gouman J, Houtman D, Vijlbrief B, Riedijk S, Verhoef P (2021). The DNA-dialogue: a broad societal dialogue about Human Germline Genome Editing in the Netherlands.

[CR49] Van Beers BC (2020) Rewriting the human genome, rewriting human rights law? Human rights, human dignity, and human germline modification in the CRISPR era. J Law Biosci 7 (1): lsaa006 10.1093/jlb/lsaa006 PMC8248990 34221419

[CR50] Van der Burg W, Brom F (2000) Legislation on ethical issues: towards an interactive paradigm. Ethical Theory and Moral Practice 3: 57-75 10.1023/a:1009987819400 15586935

[CR51] van Doorn M (2023) Waarom we beter denken dan we denken. Amersfoort: Noordboek

[CR52] Van Klink B, Van Beers B, Poort L (2016) Symbolic legislation theory and developments in biolaw. Springer

[CR53] WHO Expert Advisory Committee on Developing Global Standards for Governance and Oversight of Human Genome Editing. 2021. Human Genome Editing: recommendations. Health Ethics & Governance, World Health Organization (Geneva). https://www.who.int/publications/i/item/9789240030381.

