# Supplementary material for: The need to set explicit goals for human germline gene editing public dialogues
Source: J Community Genet. 2024 May 8;15(3):259–65. doi: 10.1007/s12687-024-00710-1 (PMC11217238; doi:10.1007/s12687-024-00710-1)
Supplement: Supplementary file 1 — Supplementary file1 (DOCX 14.3 KB) [file 12687_2024_710_MOESM1_ESM.docx]

**Supplementary Information**

**Summary of discussion of goals among the consortium**

Four goals form the foundation for how *De DNA dialogen* consortium will engage various publics and stakeholders on HGGE in the Netherlands. In three (online) meetings of approximately 1 to 1.5 hours each, these goals were discussed among the five workpackages encompassing our transdisciplinary consortium. During these meetings with about 20 attendees, including workpackage leaders and consortium members, potential goals of public engagement based on those described in the literature were discussed. These meetings were led by workpackage one (Preparation and Research). These goals of public engagement were presented to the consortium to be used as a starting point. In preparation of the discussion during the following meeting(s) each workpackage was asked to provide feedback on the goals that followed from the discussion in an online document. The intensive discussions and several rounds of written feedback resulted in the four goals as discussed in this paper.

**The ‘De DNA dialogen’ consortium**

| **Name** | **Affiliatie** |
| --- | --- |
| S.J.A.N. Arnold | Erasmus Medical Center, Department of Clinical Genetics, Rotterdam, the Netherlands |
| B. Burgers | Nemo Kennislink, Amsterdam, the Netherlands |
| M.C. Cornel | Amsterdam UMC, location Vrije Universiteit Amsterdam, Department of Human Genetics, Amsterdam, the Netherlands |
| D. Greeven | Genome Scan, Leiden, the Netherlands |
| E. Grob | Nemo Kennislink, Amsterdam, the Netherlands |
| L. Jacobs | Vrije Universiteit, Faculty of Law, Department of Legal Philosophy, Amsterdam, the Netherlands |
| B. Vijlbrief | Erasmus Medical Center, Department of Clinical Genetics, Rotterdam, the Netherlands |
| T. Vrijenhoek | University Medical Center Utrecht, Department of Medical Genetics, Utrecht, the Netherlands |
| J. Wiegertjes | Nemo Kennislink, Amsterdam, the Netherlands |
| M. Woensel, van | Nemo Kennislink, Amsterdam, the Netherlands |
